# Supplementary material for: Diagnosis of protozoa diarrhoea in Campylobacter patients increases markedly with molecular techniques
Source: PLOS Glob Public Health. 2023 May 30;3(5):e0001527. doi: 10.1371/journal.pgph.0001527 (PMC10228808; doi:10.1371/journal.pgph.0001527)
Supplement: S1 Table — Adjusted data are based only on the differences between our research laboratory and the accredited diagnostic laboratories diagnostic results for Campylobacter positive but protozoa negative test results. (DOCX) [file pgph.0001527.s001.docx]

**Supplementary information**

Table S1: The actual (left) and adjusted (right) case notifications of the most notified infectious diseases in New Zealand in 2016. Adjusted data are based only on the differences between our research laboratory and the accredited diagnostic laboratories diagnostic results for *Campylobacter* positive but protozoa negative test results.

| Disease | Notifications 2016 | Disease | Adjusted 2016 |
| --- | --- | --- | --- |
| Campylobacteriosis | 7456 | Campylobacteriosis | 7456 |
| **Giardiasis** | **1617** | **Cryptosporidiosis** | **3448** |
| Pertussis | 1096 | **Giardiasis** | **1953** |
| Salmonellosis | 1091 | Pertussis | 1096 |
| **Cryptosporidiosis** | **1062** | Salmonellosis | 1091 |
